# Phenotypic, Pot-Experimental, and Genomic Characterization of *Staphylococcus succinus* NYN-1, a Moderately Halophilic Bacterium Isolated from the Rhizosphere of the Halophyte *Suaeda dendroides* in Xinjiang

**DOI:** 10.3390/microorganisms14030680

**Published:** 2026-03-17

**Authors:** Yuxiang Huang, Jingyi Wang, Jinzhu Song, Qi Wang

**Affiliations:** 1 Xinjiang Production and Construction Corps Key Laboratory of Oasis Town and Mountain-Basin System Ecology, Ministry of Education Key Laboratory of Xinjiang Phytomedicine Resource Utilization, College of Life Sciences, Shihezi University, Shihezi 832000, China20231006110@stu.shzu.edu.cn (J.W.); 2 School of Life Science and Technology, Harbin Institute of Technology, Harbin 150080, China

**Keywords:** soil salinization, halophilic bacterium, PGPR, whole-genome analysis

## Abstract

Soil salinization is a major constraint on sustainable agriculture worldwide, highlighting the need for stress-tolerant plant growth-promoting rhizobacteria (PGPR) for salt-affected soils. A moderately halophilic and alkali-tolerant bacterium, *Staphylococcus succinus* NYN-1, was isolated from the rhizosphere soil of the halophyte *Suaeda dendroides* collected from a highly salinized site in Xinjiang, China. This study aimed to evaluate its salt–alkali tolerance and plant growth-promoting potential through integrated phenotypic characterization, pot experiments, and whole-genome analysis. NYN-1 grew over a broad salinity range [0–15% (*w*/*v*)] and pH range (6.0–11.0), and showed plant growth-promoting activities including organic phosphorus mineralization, inorganic phosphate solubilization, potassium solubilization, and NH_4_^+^ production. In pot experiments under 300 mM NaCl, inoculation with NYN-1 significantly improved the growth performance of maize (*Zea mays* L.), cotton (*Gossypium hirsutum* L.), and sunflower (*Helianthus annuus* L.). Genome analysis identified multiple Na^+^/H^+^ antiporter-related genes and genes encoding compatible-solute transport systems that are consistent with adaptation to salt–alkali stress. The genome also harbors a broad set of genes related to phosphorus metabolism, as well as other plant growth-promoting functions, including potassium solubilization-related pathways and siderophore biosynthesis. Collectively, these findings identify *S. succinus* NYN-1 as a promising native halophilic PGPR candidate and a potential microbial resource for developing inoculant strategies in salt-affected agricultural systems.

## 1. Introduction

Soil salinization is one of the major environmental problems constraining the development of global sustainable agriculture [1,2]. Soluble salts accumulated in soil adversely affect crop growth primarily through osmotic stress and ion toxicity [3], ultimately reducing crop yield and quality. According to the 2024 global report on salt-affected soils released by the Food and Agriculture Organization, the area of salt-affected land worldwide has exceeded 1.381 billion hectares, accounting for approximately 10.7% of the global land surface [4]. With ongoing climate change, the proportion of this land type is expected to further expand, highlighting the importance of developing environmentally friendly and sustainable management strategies for saline–alkaline land management and improvement.

Currently, saline–alkaline land improvement measures can be classified into four main types: physical, hydraulic, chemical, and biological approaches. Biological remediation is particularly important due to its low cost and ecological benefits. Common biological strategies include the use of plants, especially salt-tolerant or salt-accumulating species, to absorb soil salts or sodium ions [5]. Another strategy involves applying microorganisms, typically as microbial fertilizers or inoculants, alone or in combination with chemical fertilizers [6]. Plant growth-promoting rhizobacteria are a group of microorganisms that have been extensively studied and widely applied to enhance plant salt tolerance and promote nutrient uptake in the rhizosphere [7,8,9]. Studies have demonstrated that PGPR from various genera can promote plant growth through multiple mechanisms, such as solubilizing insoluble mineral nutrients [10], synthesizing plant hormones or their precursors [11], and regulating plant antioxidant enzyme activities [12]. However, the effectiveness of PGPR in soil is often limited by harsh environmental conditions, including pH and salinity [13], underscoring the need to develop PGPR that can adapt to saline–alkaline environments, persist long term, and consistently exert growth-promoting functions.

Halophilic microorganisms are a group of microbes capable of surviving in environments with high salt concentrations, with optimal growth at salinities above 0.2 M [14]. They maintain cellular osmotic balance primarily through two mechanisms: the salt-in strategy [15] and the accumulation of compatible solutes [16]. These microorganisms possess significant application potential in the food industry, the pharmaceutical field, and saline–alkaline land remediation [14].

Northwestern China, particularly the Xinjiang region, contains extensive areas of saline–alkaline soils and represents one of the world’s typical arid saline–alkaline ecosystems. While large expanses of saline–alkaline land hinder local agricultural development, they also serve as reservoirs of halophilic microbial resources with strong saline–alkaline tolerance and environmental adaptability. Recent studies indicate that native core microorganisms are more likely than exogenous strains to stably colonize target habitats and maintain their functions [17]. Plants can selectively enrich native microorganisms that benefit their growth under abiotic stress [18], suggesting that native halophilic microorganisms may have significant potential for applications in saline–alkaline agriculture and ecological restoration.

The bacterium *Staphylococcus succinus*, a Gram-positive species, has previously been studied for its role in food fermentation processes, including enhancing the flavor of fermented chili [19] and facilitating the production of volatile compounds in soybeans [20]. Recent studies have identified the potential of *S. succinus* strains as PGPR, with demonstrated abilities such as enhancing zinc uptake and oil content in rapeseed [21] and promoting wheat seedling growth under salt stress [22]. However, research on the adaptation mechanisms of S. succinus to saline–alkali environments and its plant growth-promoting functions remains limited, especially regarding functional genomic analyses.

In this context, we investigated strain NYN-1, an indigenous, moderately halophilic and alkali-tolerant bacterium identified as *Staphylococcus succinus* and isolated from the rhizosphere of the halophyte *Suaeda dendroides* in Xinjiang. By integrating salt-tolerance assays, plant growth-promoting trait screening, and whole-genome sequencing, this study systematically evaluated the strain’s adaptive characteristics and plant growth-promoting potential under salt stress. The findings provide a theoretical basis for the utilization of indigenous halophilic microbial resources and support the development of microbial strategies for sustainable improvement of saline–alkali soils, particularly in Xinjiang.

## 2. Materials and Methods

### 2.1. Isolation of the Plant Growth-Promoting Rhizobacterium S. succinus NYN-1

Strain NYN-1 was isolated from the rhizosphere soil of the halophyte *Suaeda dendroides* at Jiahezi Reservoir, Manas County, Changji Hui Autonomous Prefecture, Xinjiang Uygur Autonomous Region, China (44°27′25″ N, 86°7′38″ E). The sampling site is a highly salinized habitat where *S. dendroides* is a dominant halophytic species, providing a suitable ecological niche for salt-tolerant and alkali-tolerant microorganisms. According to the World Reference Base for Soil Resources (WRB) classification system, soils in this region are typically classified as Solonchaks, which are characterized by high concentrations of soluble salts. Plants of *S. dendroides* showing uniform growth were selected and carefully uprooted. Soil tightly adhering to the roots (approximately 5 mm thickness) was collected using sterile gloves and sterile forceps, transferred into sterile plastic bags, preserved in liquid nitrogen immediately after collection, and promptly transported to the laboratory. Soil moisture and ambient temperature at the sampling site were not quantitatively recorded at the time of collection.

A 5 g sample of rhizospheric soil was inoculated into a conical flask containing 100 mL of sterile LB broth supplemented with 50 g/L NaCl, sealed with parafilm, and incubated in a shaker at 28 °C and 180 rpm for a 24 h enrichment culture. The resulting bacterial suspension was serially diluted, and 100 µL aliquots of the 10^−2^, 10^−3^, 10^−4^, and 10^−5^ dilutions were spread onto sterile LB agar plates containing 50 g/L NaCl using the spread plate method. Plates were incubated in an inverted position at 28 °C for 48 h to obtain single colonies. Colonies with distinct morphology were selected and purified by repeated streaking. Candidate isolates were then subjected to preliminary screening for salt tolerance in sterile LB broth supplemented with 50 g/L NaCl, followed by initial screening for plant growth-promoting traits, including nitrogen fixation, phosphorus solubilization, and potassium solubilization. Based on these preliminary screenings, strain NYN-1 was selected for further study.

### 2.2. Determination of Salt and Alkali Tolerance in NYN-1

A 400 μL aliquot of the glycerol stock stored at −80 °C was inoculated into 40 mL of sterile LB broth and incubated in a standard temperature-controlled shaker at 28 °C with shaking at 130 rpm until the optical density at 600 nm (OD_600_) reached 1.0. No specific photoperiod was controlled during incubation. Culture vessels were sealed with standard rubber stoppers, and no additional aeration control was applied.

Sterile LB broth was adjusted (using HCl and NaOH) to pH 4.0, 5.0, 6.0, 7.0, 8.0, 9.0, 10.0, 11.0, and 12.0, autoclaved at 121 °C for 20 min, and cooled before use. The pre-culture was inoculated at 0.25% (*v*/*v*) into sterile LB broth with the indicated pH values and incubated in the same temperature-controlled shaker at 28 °C with shaking at 130 rpm until the stationary phase. The OD_600_ was then measured and recorded.

Sterile LB broth was supplemented with NaCl at 0, 0.5%, 1%, 3%, 5%, 7%, 10%, 12%, 15%, 18%, 20%, 23%, and 25% (*w*/*v*), autoclaved at 121 °C for 20 min, and cooled before use. The strain was inoculated at the same ratio into sterile LB broth with the indicated NaCl concentrations and incubated in the same temperature-controlled shaker at 28 °C with shaking at 130 rpm until the stationary phase. The OD_600_ was then measured and recorded. All experiments were conducted in triplicate.

### 2.3. Detection of Plant Growth-Promoting Activities of NYN-1

The following assays were performed as preliminary screening tests for plant growth-promoting traits. All assays were conducted with three independent biological replicates.

#### 2.3.1. Nitrogen Fixation, Phosphorus Solubilization, and Potassium Solubilization

Single colonies of the previously isolated and purified NYN-1 strain were individually inoculated onto sterile agar plates containing Ashby’s nitrogen-free medium [23], inorganic phosphate-solubilizing bacterial screening medium [24], organic phosphate-solubilizing bacterial screening medium [25], and potassium-solubilizing bacterial screening medium [26]. The plates were incubated in an inverted position at 28 °C for 5 days. The growth status of the strain and the size and color of transparent halos formed on the media were observed to assess its nitrogen-fixing, phosphorus-solubilizing, and potassium-solubilizing plant growth-promoting activities. Post-incubation pH of the media was not measured in these plate-based screening assays.

#### 2.3.2. NH_4_^+^ Production

A single colony of the isolated and purified strain was inoculated into sterile peptone water and incubated at 28 °C for 48 h on a rotary shaker at 180 rpm. The culture broth was mixed with Nessler’s reagent at a 1:1 volume ratio, and the OD_530_ was measured using a spectrophotometer. An OD_530_ value greater than 0.1 was considered positive compared with the negative control, and higher OD_530_ values were interpreted as indicating relatively stronger NH_4_^+^ production in this screening assay [27]. No external calibration standard or standard curve was used in this assay.

#### 2.3.3. Indole-3-Acetic Acid (IAA) Production

The strain was inoculated into sterile LB broth supplemented with 100 mg/L L-tryptophan and incubated on a rotary shaker at 28 °C and 180 rpm for 3 days. After centrifugation at 8000 rpm, 50 μL of the culture supernatant was mixed with 50 μL of Salkowski reagent and spotted onto a white porcelain plate. After incubation in the dark for 30 min, pink coloration was considered positive, indicating that the strain could secrete IAA. Deeper coloration was interpreted as indicating relatively stronger IAA production in this qualitative assay, while no color change was considered negative, indicating the inability to produce IAA [28]. No IAA calibration standard or standard curve was used.

#### 2.3.4. ACC Deaminase Production

A single colony was cultured in sterile LB broth and incubated at 28 °C and 180 rpm until the logarithmic growth phase was reached. The cells were collected by centrifugation, washed twice with 0.1 M Tris-HCl buffer (pH 7.6), and centrifuged at 10,000 rpm for 5 min; the supernatant was discarded. The cell pellet was resuspended in 600 μL of 0.1 M Tris-HCl buffer (pH 8.5), followed by the addition of 30 μL toluene and vigorous mixing for 30 s. An aliquot of 200 μL was transferred into a 1.5 mL centrifuge tube, mixed with 200 μL of 0.5 M ACC for 5 s, and incubated in a 30 °C water bath for 15 min. Subsequently, 0.56 M HCl was added, the mixture was mixed thoroughly, and it was centrifuged at 10,000 rpm for 5 min at room temperature. One milliliter of the supernatant was mixed with 800 μL of 0.56 M HCl in a test tube, followed by the addition of 300 μL of 2,4-dinitrophenylhydrazine, and incubated at 30 °C for 30 min. After the addition of 2 M NaOH, the OD_450_ was measured, and values clearly higher than those of the negative control indicated that the strain exhibited ACC deaminase activity [29].

### 2.4. Pot Experiment with Maize, Sunflower, and Cotton

Maize, cotton, and sunflower were selected because they represent agronomically important crop types in Xinjiang, including a major grain crop, a major cash crop, and an oilseed crop, respectively. In addition, these crops differ in root system architecture, with maize representing a fibrous-rooted monocot and cotton and sunflower representing tap-rooted dicots, allowing a preliminary evaluation of the cross-crop applicability of strain NYN-1.

#### 2.4.1. Seed Treatment

In this experiment, seeds of maize (XinYu 108, Jiushenghe Seed Industry Co., Ltd., Changji, China), sunflower (AiDaTou 567DW, China National Seed Group Co., Ltd., Sanya, China), and cotton (XinLuZao 84, HeXin Technology Development Co., Ltd., Shihezi, China) were used. Uniform and fully developed maize, sunflower, and cotton seeds were selected and surface-sterilized by soaking in 10% (*v*/*v*) sodium hypochlorite solution for 10 min, followed by rinsing with sterile water at least three times until no residual chlorine odor remained. The sterilized seeds were then placed on sterile Petri dishes lined with gauze moistened with sterile water and germinated at 25 °C for 24 h prior to use.

#### 2.4.2. Pot Experiment

Maize, cotton, and sunflower seeds were first germinated in plug trays (cell size: 60 mm × 60 mm) containing sterilized nutrient soil, vermiculite, and perlite mixed at a 1:1:1 ratio. One seed was sown per tray cell. When seedlings reached approximately 3–4 cm in height, uniform seedlings were selected and transplanted into PP pots (13 cm in diameter and 10 cm in height) filled with the same substrate mixture, with two plants maintained per pot.

Each pot contained approximately 0.8 kg of substrate consisting of sterilized nutrient soil, vermiculite, and perlite (1:1:1). A non-NaCl control (0 mM NaCl) was included in the experimental design. Soil salinity in the salt-stress treatment was maintained by applying 300 mM NaCl solution every 3 days. This concentration was selected based on preliminary laboratory experiments testing NaCl levels between 100 and 300 mM, which indicated that 300 mM NaCl significantly inhibited plant growth while still allowing plant survival, thereby providing an appropriate salt-stress level for evaluating the plant growth-promoting effects of strain NYN-1.

The bacterial suspension was applied by root drenching every 7 days at 50 mL per pot, and the inoculum was prepared as a bacterial suspension adjusted to OD_600_ = 0.6.

Plants were grown under a 12 h light/12 h dark photoperiod at room temperature (approximately 25 °C) for 30 days. The pH of the substrate was not instrumentally measured during the experiment; however, the commercial nutrient soil used in the substrate typically has a pH range of approximately 6.0–7.5. This pH range is commonly considered suitable for the growth of maize, cotton, and sunflower seedlings. Light intensity and relative humidity were not instrumentally monitored during the experiment.

Each treatment included three biological replicates, resulting in a total of twelve pots per plant species. At the end of the experiment, plants were harvested for measurement of root length, plant height, fresh weight, dry weight, and other growth-related parameters.

#### 2.4.3. Determination of Plant Physiological and Biochemical Parameters

For each assay, 0.1 g of leaf tissue was homogenized on ice using a pre-chilled mortar. The homogenate was extracted using the extraction buffer provided in the corresponding assay kit, following the manufacturer’s instructions. Catalase (CAT) activity and malondialdehyde (MDA) content in plant leaf tissues were then measured using the corresponding assay kits (spectrophotometric method) obtained from Addison Biotechnology Co., Ltd. (Yancheng, China).

### 2.5. Genome Sequencing and Annotation

#### 2.5.1. Genome Sequencing and Functional Annotation

Genomic DNA of strain NYN-1 was extracted using the FastPure Bacteria DNA Isolation Mini Kit (Vazyme Biotech Co., Ltd., Nanjing, China). Sequencing was conducted by Shanghai Majorbio Bio-Pharm Technology Co., Ltd. (Shanghai, China). using the PacBio Sequel (Pacific Biosciences (PacBio), Menlo Park, CA, USA.) and Illumina NovaSeq PE150 platforms (Illumina, Inc., San Diego, CA, USA.). Low-quality reads were filtered with fastp v0.23.0 to ensure data integrity and generate high-quality overlapping sequences. Gene annotation and functional prediction utilized the GeneMarkS, Pfam, Swiss-Prot, NR, GO, KEGG, and COG databases. Data visualization was performed using the Majorbio Cloud Platform (https://cloud.majorbio.com, accessed on 21 January 2025) [30]. The complete genome sequence and the 16S rRNA gene sequence of strain NYN-1 have been deposited in the NCBI GenBank database under the accession numbers JBTWDY000000000.1 and PX468777.1, respectively.

#### 2.5.2. Average Nucleotide Identity (ANI) Analysis

The Average Nucleotide Identity (ANI) between strain NYN-1 and 19 closely related bacterial genomes was calculated to confirm its taxonomic position. The analysis was performed using FastANI (v1.32) software on the Majorbio Cloud Platform (https://cloud.majorbio.com, accessed on 21 January 2025). The genomic sequences were fragmented into 3000 bp pieces for reciprocal alignment, and the resulting identity percentages were used to generate the ANI heat map.

### 2.6. Statistical Analysis and Figure Preparation

Statistical analyses were performed using SPSS 19.0 (IBM Corp., Armonk, NY, USA). Data normality was assessed using the Shapiro–Wilk test prior to one-way analysis of variance (ANOVA). One-way ANOVA followed by Tukey’s honestly significant difference (HSD) test was used to determine significant differences among treatments at *p* < 0.05. Data are presented as mean ± SD. All quantitative graphs were generated using Origin 2021 (OriginLab Corp., Northampton, MA, USA), whereas all schematic illustrations were created using BioRender (https://app.biorender.com, accessed on 21 January 2025). Phylogenetic trees were constructed using MEGA 11 software. The Neighbor-Joining (NJ) method based on the Maximum Composite Likelihood model was employed to estimate evolutionary distances.

## 3. Results

### 3.1. Basic Characteristics of S. succinus NYN-1

**Figure 1:**
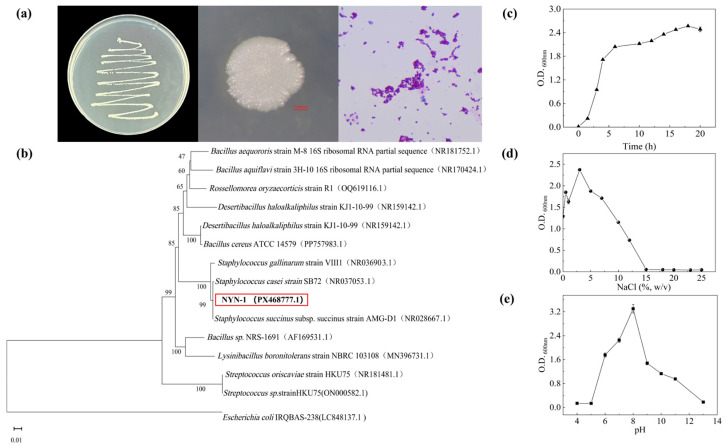
Basic characteristics of *S. succinus* NYN-1. (**a**) Colony morphology on sterile LB agar plates (**left**), a single-colony morphology under stereomicroscope (**middle**), and Gram-staining cells (**right**); (**b**) 16S rRNA gene-based phylogenetic tree of strain NYN-1 constructed using the neighbor-joining method; bootstrap values (%) are shown at nodes; (**c**) Growth curve of the strain; (**d**) salinity tolerance range of the strain; (**e**) pH tolerance range of the strain. The strain NYN-1 highlighted in the red box is the subject of this study (GenBank accession number for the 16S rRNA sequence: PX468777.1).

Colonies of strain NYN-1 were milky white, opaque, nearly circular, with irregular margins under stereomicroscopic observation. Gram staining confirmed that the strain was Gram-positive (Figure 1a). In the 16S rRNA gene-based phylogenetic tree constructed using the neighbor-joining method, NYN-1 formed a sister lineage with *Staphylococcus succinus* AMG-D1 (bootstrap = 99), sharing 99% 16S rRNA gene sequence similarity (Figure 1b). These results suggested that NYN-1 was affiliated with the genus *Staphylococcus*.

Growth and tolerance assays showed that NYN-1 reached the stationary phase by approximately 18 h after inoculation, with a salinity tolerance range of 0–15% (*w*/*v*) and an optimal salinity of about 3% (*w*/*v*) (Figure 1c,d). NYN-1 also grew across pH 6.0–11.0, with an optimum at approximately pH 8.0 (Figure 1e). Overall, according to the definitions of halophilic and alkali-tolerant microorganisms [14,31], NYN-1 was identified as a moderately halophilic and alkali-tolerant strain.

### 3.2. Plant Growth-Promoting Activities of S. succinus NYN-1

**Figure 2:**
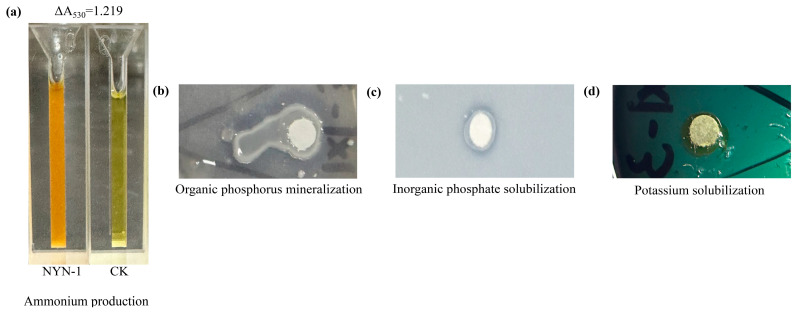
Qualitative screening of plant growth-promoting activities of *S. succinus* NYN-1. (**a**) NH_4_^+^ production; (**b**) Organic phosphorus mineralization; (**c**) Inorganic phosphorus solubilization; (**d**) Potassium solubilization.

NYN-1 was positive for multiple plant growth-promoting traits in qualitative screening assays (Table 1). After 48 h of cultivation in peptone water, the culture mixed with Nessler’s reagent at a 1:1 volume ratio showed a ΔA530 of 1.219 relative to the control (CK), consistent with NH_4_^+^ production (Figure 2a). Clear halo zones were observed on both organic phosphorus mineralization medium and inorganic phosphate-solubilizing medium, indicating a phosphorus-mobilizing phenotype (Figure 2b,c). On potassium-solubilizing medium, the inoculation zone of strain NYN-1 changed from green to yellow, consistent with potassium solubilization (Figure 2d). In contrast, nitrogen fixation, IAA production, and ACC deaminase activity were not detected under the conditions tested (Table 1).

### 3.3. Growth-Promoting Effects of S. succinus NYN-1 on Maize, Cotton, and Sunflower Under Salt Stress

**Figure 3:**
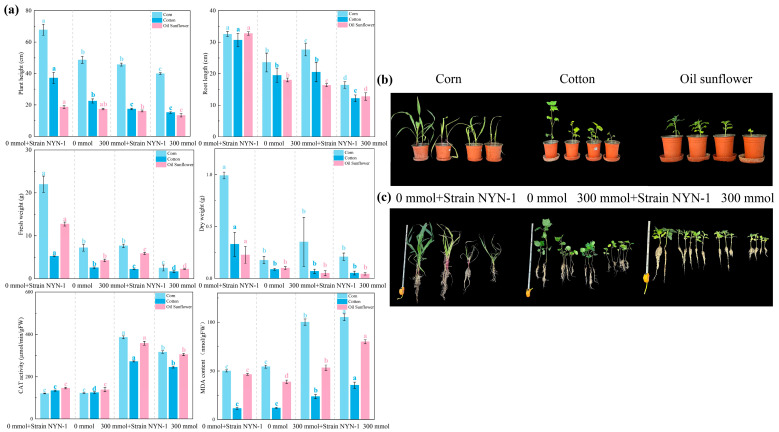
Plant growth-promoting effects of *S. succinus* NYN-1 (**a**) plant height, root length, fresh weight, dry weight, CAT activity, and MDA content; (**b**) Pot experiment photographs; (**c**) Plant photographs. According to the HSD test, different letters above the bars indicate significant differences at *p* ≤ 0.05.

Overall, 300 mM NaCl stress inhibited the growth of maize, cotton, and sunflower, as reflected by reduced plant height, root length, and fresh and dry biomass (Figure 3a–c). NYN-1 inoculation partially mitigated salt-induced growth inhibition, with effects differing among crops and traits.

In maize, NYN-1 inoculation significantly increased plant height, root length, and biomass under non-saline conditions (Figure 3a). Under 300 mM NaCl stress, NYN-1 inoculation significantly increased root length and fresh weight by 68.2% and 209.6%, respectively, relative to the salt-stressed control (Figure 3a). NYN-1 inoculation also significantly improved other growth traits and increased leaf CAT activity under salt stress (Figure 3a).

In cotton, NYN-1 inoculation significantly increased plant height and biomass under non-saline conditions, whereas root length did not change significantly (Figure 3a). Under salt stress, NYN-1 inoculation significantly increased root length and fresh weight by 187.4% and 35.7%, respectively, compared with the salt-stressed control (Figure 3a). In addition, NYN-1 inoculation significantly increased leaf CAT activity and decreased MDA content under salt stress (Figure 3a).

In sunflower, NYN-1 inoculation significantly improved root length and biomass under both non-saline and salt-stress conditions (Figure 3a). Under 300 mM NaCl stress, NYN-1 inoculation significantly increased root length and fresh weight by 81.9% and 198.6%, respectively, relative to the salt-stressed control (Figure 3a). NYN-1 inoculation also significantly increased leaf CAT activity and decreased MDA content under salt stress (Figure 3a).

### 3.4. Genome Features and Functional Annotation of S. succinus NYN-1

**Figure 4:**
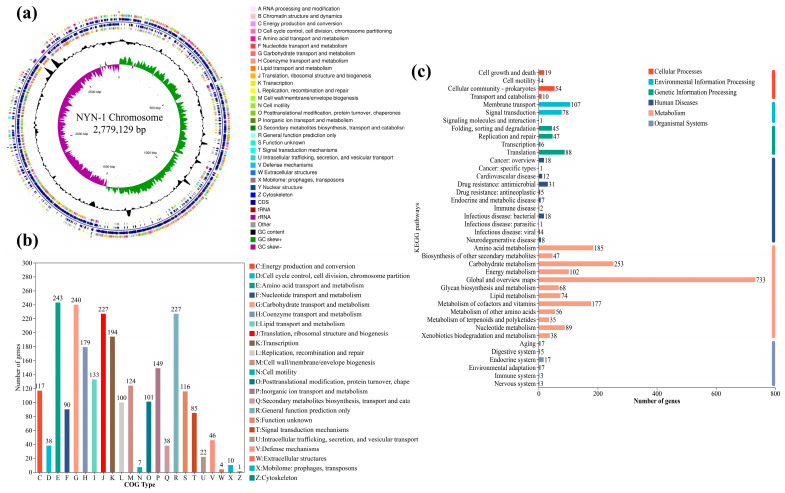
Genome features and functional annotation of *S. succinus* NYN-1 (**a**) Circular genome map; (**b**) Functional annotation based on the COG database; (**c**) Functional annotation based on the KEGG database.

The NYN-1 genome was 2,779,129 bp in length, with a GC content of 31.6%, and no plasmids were detected. Genome annotation identified 2594 protein-coding sequences, 55 tRNA genes, 25 rRNA genes, 45 sRNA genes, and 36 tandem repeats (Figure 4a).

COG annotation showed that genes were mainly assigned to categories related to amino acid transport and metabolism, carbohydrate transport and metabolism, translation, ribosomal structure and biogenesis, and general function prediction, suggesting broad functional diversity in strain NYN-1 (Figure 4b). KEGG annotation showed that genes assigned to metabolic pathways were particularly enriched in carbohydrate metabolism (253 genes), amino acid metabolism (185 genes), metabolism of cofactors and vitamins (177 genes), and energy metabolism (102 genes), indicating strong metabolic versatility in strain NYN-1 (Figure 4c).

### 3.5. Taxonomic Position of S. succinus NYN-1

**Figure 5:**
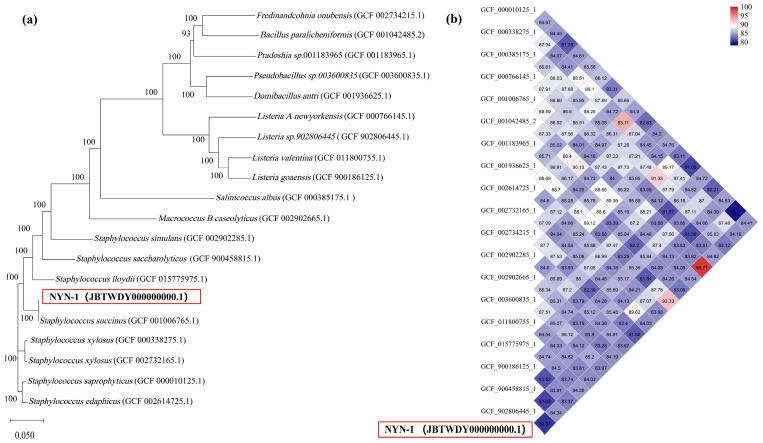
Taxonomic position of *S. succinus* NYN-1 (**a**) Neighbor-joining phylogenetic tree constructed from concatenated sequences of 31 housekeeping genes extracted from NYN-1 and 19 closely related genomes; bootstrap support values (%) are shown at nodes; (**b**) Pairwise ANI heatmap comparing strain NYN-1 with 19 closely related genomes. The red boxes in both panels highlight the strain *Staphylococcus succinus* NYN-1 investigated in this study. The alphanumeric code in parentheses (JBTWDY000000000.1) refers to its GenBank whole-genome sequence accession number.

A phylogenetic tree was constructed using concatenated sequences of 31 conserved housekeeping genes extracted from NYN-1 and 19 closely related genomes (Figure 5a). In this phylogeny, NYN-1 clustered with *S*. *succinus* DSM 14617 (GCF 001006765.1) with strong bootstrap support. Pairwise ANI analysis further showed that NYN-1 shared an ANI of 98.28% with DSM 14617, exceeding the commonly used species boundary (95%), whereas ANI values between NYN-1 and the remaining genomes were all below 95% (Figure 5b). Collectively, these results support the assignment of strain NYN-1 to the species *Staphylococcus succinus*.

### 3.6. Growth-Promoting Genes Associated with S. succinus NYN-1

#### 3.6.1. Phosphorus Solubilization

**Figure 6:**
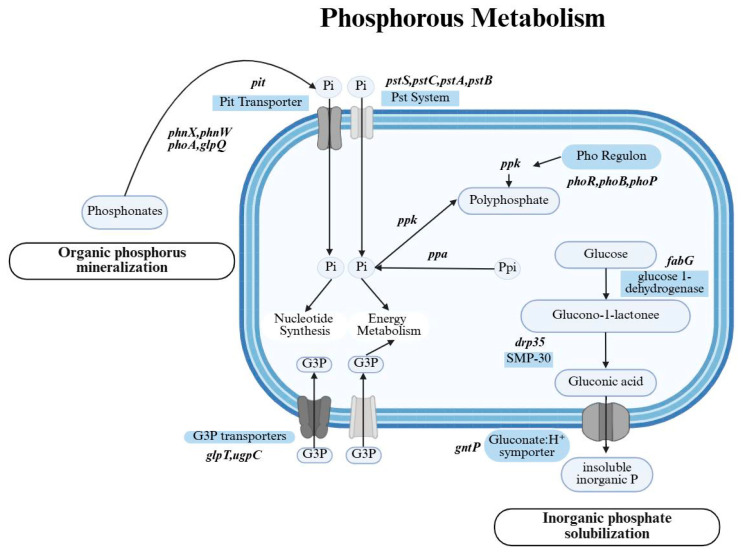
Schematic overview of predicted phosphorus metabolism and cycling pathways in *S. succinus* NYN-1 based on genome annotation.

Strain NYN-1 showed phosphate-mobilizing phenotypes in the qualitative screening assays, including inorganic phosphate solubilization and organic phosphorus mineralization. PCycDB-based annotation identified 67 phosphorus metabolism-related genes in the NYN-1 genome [32]. Of these, 26 genes were assigned to three core functional categories defined in PCycDB: phosphorus activation, phosphorus uptake, and regulation of the phosphate starvation response (Table 2) [32]. The remaining genes were assigned to other metabolic categories. An annotation-based schematic summary of the predicted phosphorus-related pathways in NYN-1 is presented in Figure 6.

Genes annotated as related to organic phosphorus utilization were identified, including *phoA* (alkaline phosphatase) and *glpQ* (glycerophosphodiester phosphodiesterase) [33,34,35]. Genes associated with phosphonate utilization, including *phnW* and *phnX*, were also detected [36]. The *pqqA–pqqF* gene cluster was not identified in the NYN-1 genome [37,38]. Genes related to gluconate transport and metabolism, including *gntP*, *gntK*, and gnd, were present [39].

The NYN-1 genome harbored genes encoding both high-affinity and low-affinity phosphate transport systems, including the pstSCAB operon and *pit* [40,41]. Genes encoding transporters related to glycerol-3-phosphate uptake, including *glpT* and *ugpC*, were also identified [42,43]. Genes associated with phosphate homeostasis were also detected, including *ppk* and *ppa* [44,45,46]. Genes annotated as components of phosphate-starvation regulation, including *phoB*, *phoR*, *phoU*, *phoH*, and *phoP*, were also present in the NYN-1 genome [47].

#### 3.6.2. Ammonium Production and Potassium Solubilization

Nitrogen metabolism-related genes annotated in the NYN-1 genome were limited (Table 3). KEGG-based annotation did not identify nitrogenase-encoding genes in the NYN-1 genome. In addition, *gudB*, annotated as encoding glutamate dehydrogenase, was identified [48]. Genes associated with assimilatory or dissimilatory nitrate reduction were not detected in the NYN-1 genome.

Additionally, the NYN-1 genome harbors *ackA* and *mdh*, encoding acetate kinase and malate dehydrogenase, respectively. These genes were annotated as related to acetate- and malate-associated metabolic pathways [49,50].

### 3.7. Saline–Alkaline Tolerance-Related Genes of S. succinus NYN-1

**Figure 7:**
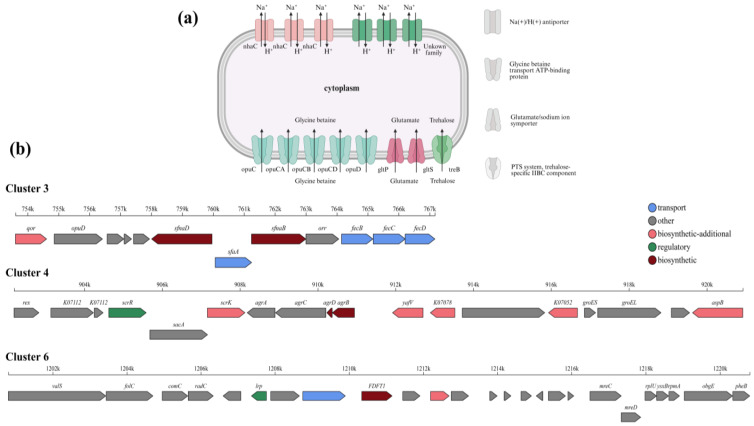
Saline–alkaline tolerance and secondary metabolic characteristics of strain NYN-1 from a genomic perspective. (**a**) Saline–alkaline tolerance-related transporters annotated in the NYN-1 genome based on the TCDB database; (**b**) Chromosomal organization of saline–alkaline tolerance and plant growth-promoting secondary metabolite gene clusters in strain NYN-1, showing all predicted genes within each cluster, with different colors representing functional categories of genes involved in secondary metabolite biosynthesis.

The *S. succinus* NYN-1 strain was isolated from the rhizosphere soil of the halophytic plant *Suaeda dendroides*. Experimental results demonstrated that this strain can grow under NaCl concentrations ranging from 0 to 15% and pH values from 6.0 to 11.0 (Figure 1d,e). The optimal salinity and pH were approximately 3% NaCl and 8.0, respectively, indicating that NYN-1 is a moderately halophilic and alkali-tolerant bacterium. Annotation against the TCDB database revealed multiple genomic features potentially associated with saline–alkaline tolerance in the NYN-1 genome (Figure 7a).

#### 3.7.1. Cation/Proton Antiporters

Based on TCDB annotation, multiple Na^+^/H^+^ antiporter-related genes were identified in the NYN-1 genome, including three NhaC family Na^+^/H^+^ antiporters and additional putative Na^+^/H^+^ antiporter components that could not be assigned to a specific family (Table 4). In contrast, genes annotated as K^+^/H^+^ or Ca^2+^/H^+^ antiporters were not detected in NYN-1 (Table 4).

#### 3.7.2. Transport of Compatible Solutes

Genes associated with uptake of compatible solutes were identified in the NYN-1 genome (Table 4). For glycine betaine, multiple opu genes (*opuA*, *opuB*/*opuD*, *opuC*, and *opuD*) were annotated [51]. For glutamate, one *gltP* and one *gltS* gene were identified and annotated as proton/glutamate symporters [52]. In addition, *treB*, annotated as a trehalose-specific phosphotransferase system (PTS) component, was detected [53].

### 3.8. Secondary Metabolite Biosynthetic Gene Clusters of S. succinus NYN-1

antiSMASH analysis identified six predicted biosynthetic gene clusters (BGCs) in the NYN-1 genome, including one NRPS cluster (cluster 1), one type III polyketide synthase (T3PKS) cluster (cluster 2), one siderophore cluster (cluster 3), one cyclic-lactone autoinducer-associated cluster (cluster 4), and two terpene clusters (clusters 5 and 6) (Table 5). Among these, the siderophore cluster (cluster 3) showed 100% similarity to the staphyloferrin A BGC in MIBiG (BGC0000944), whereas clusters 2 and 4 showed only low similarity to their closest MIBiG matches (3–4%).

Cluster 3 was predicted as a siderophore biosynthetic gene cluster and showed 100% similarity to the staphyloferrin A biosynthetic gene cluster in MIBiG (BGC0000944). This cluster contains *iucA*/*iucC* family genes annotated as siderophore biosynthesis proteins and genes encoding putative ferric–siderophore uptake components [54].

A locus annotated by antiSMASH as a cyclic-lactone autoinducer-associated gene cluster (cluster 4) was identified in the NYN-1 genome (Figure 7b; Table 5) [55]. This cluster showed only low similarity (4%) to the closest MIBiG match, indicating that its predicted function should be interpreted cautiously.

Cluster 6 was predicted by antiSMASH as a terpene biosynthetic gene cluster, with core genes annotated as enzymes involved in squalene and carotenoid biosynthesis/precursor formation (Figure 7b).

## 4. Discussion

Soil salinization remains a major constraint on sustainable agriculture, underscoring the need for microbial remediation strategies that can persist and function under high salinity and alkaline pH. Notably, arid regions such as Xinjiang in northwestern China contain extensive saline–alkaline soils and represent underexploited reservoirs of native halophilic microorganisms that are pre-adapted to these environments. In this study, we isolated an indigenous, moderately halophilic and alkali-tolerant plant growth-promoting rhizobacterium, *Staphylococcus succinus* NYN-1, from the rhizosphere of the halophyte *Suaeda dendroides*. Phenotypic assays showed that NYN-1 tolerated a wide range of salinity and pH (Figure 1), and pot experiments further supported its ability to enhance crop growth under salt stress (Figure 3). By integrating these observations with whole-genome annotation, we provide a preliminary genomic framework that links saline–alkaline tolerance-related transport systems and plant growth-promoting functions in NYN-1 (Figure 7; Table 4). However, because genome-based inference reflects genetic potential rather than verified activity, the mechanistic interpretations proposed below should be considered putative and warrant further functional validation. Additionally, although NaCl was used to simulate salt stress in the pot experiment, future studies should evaluate the performance of strain NYN-1 under combined saline–alkaline conditions, such as soils containing alkaline salts (e.g., Na_2_CO_3_ or NaHCO_3_).

### 4.1. Saline–Alkaline Tolerance Characteristics of S. succinus NYN-1 from a Genomic Perspective

Phenotypic assays showed that NYN-1 tolerated a broad range of salinity and pH, with optimal growth at approximately 3% (*w*/*v*) NaCl and pH 8.0 (Figure 1d,e). Such a phenotype is consistent with the ecological advantage often attributed to moderately halophilic bacteria, which are reported to maintain growth across relatively wide salinity windows compared with more specialized halophiles [56,57]. In salt-affected soils, this physiological flexibility may be particularly valuable for rhizosphere inoculants because salinity and pH can fluctuate substantially over time and space. Mechanistically, halophilic microorganisms are generally thought to cope with osmotic and ionic stress via two non-mutually exclusive strategies: intracellular ion accumulation (“salt-in”) and the uptake or synthesis of compatible solutes [15,16]. Below, we discuss how the transporter repertoire annotated in the NYN-1 genome (Table 4; Figure 7a) is consistent with these putative strategies, while noting that genome-based predictions require functional validation.

The “salt-in” strategy is typically characterized by ion-based osmoregulation coupled with active extrusion of cytotoxic Na^+^ [58]. In many halophiles, cells counterbalance external osmolarity by accumulating intracellular K^+^ while maintaining low intracellular Na^+^ through Na^+^ efflux systems, including Na^+^/H^+^ antiporters and related transporters [14,59]. Cation/proton antiporters are commonly implicated in bacterial ion and pH homeostasis under saline–alkaline stress [60,61]. Consistent with this framework, TCDB-based annotation identified multiple Na^+^/H^+^ antiporter-related genes/components in the NYN-1 genome (Table 4; Figure 7a), whereas no K^+^/H^+^ or Ca^2+^/H^+^ antiporters were detected in this annotation. Although gene presence does not confirm activity, these features suggest that Na^+^/H^+^ exchange may contribute to ion homeostasis in NYN-1 under saline–alkaline stress, consistent with its growth across 0–15% (*w*/*v*) NaCl and pH 6.0–11.0 (Figure 1d,e).

Compatible-solute accumulation is a common microbial strategy for coping with osmotic stress because intracellular levels of these highly water-soluble, non-disruptive small molecules can be adjusted in response to changes in external salinity [58,62]. In the NYN-1 genome, TCDB-based annotation identified transporter genes related to the uptake of several common osmoprotectants, including glycine betaine, glutamate, and trehalose (Table 4; Figure 7a). Among these, multiple loci annotated as glycine betaine transport systems (e.g., Opu-type transporters) were detected, suggesting that betaine uptake may contribute importantly to osmoprotection in NYN-1. Similar betaine transport systems have been linked to osmotic tolerance in other bacteria [63], although functional assays will be required to determine their regulation and quantitative contribution in NYN-1. In addition, antiSMASH predicted a terpene biosynthetic gene cluster (cluster 6) with annotations related to carotenoid-associated metabolism (Table 5; Figure 7b). Carotenoid-type terpenoids have been reported to contribute to cellular protection against abiotic stresses in diverse bacteria, partly through membrane stabilization and mitigation of oxidative damage [64]. Accordingly, this terpene gene cluster may represent an additional, but as yet unverified, component of stress adaptation in NYN-1 under salt–alkali conditions.

### 4.2. Plant Growth-Promoting Potential of S. succinus NYN-1 from a Genomic Perspective

PGPR are beneficial microorganisms inhabiting the rhizosphere that promote plant growth and health [65]. Rhizosphere microorganisms can directly benefit crops by improving nutrient availability and secreting growth-regulating substances [66]. Functional medium assays showed that strain NYN-1 exhibits NH_4_^+^ production, organic phosphorus mineralization, inorganic phosphorus solubilization, and potassium solubilization activities, indicating its potential as a beneficial PGPR (Table 1; Figure 2). Pot experiments under 300 mM salt stress further showed that inoculation with NYN-1 displayed strong growth-promoting effects across different crop species under both non-saline and saline conditions. Under non-saline conditions, inoculation with NYN-1 significantly increased crop biomass. Under salt stress, the growth-promoting effects of NYN-1 were slightly reduced, but root length increases being more evident in most cases (Figure 3). In conclusion, *S. succinus* NYN-1 represents an effective PGPR with promising application potential for promoting the growth of multiple crops in saline–alkaline soils.

Phosphorus is an essential macronutrient for plant growth. However, in most soils, only a small fraction of total P is readily available to plants because it is largely present in insoluble mineral forms or complex organic pools [67]. Phosphate-solubilizing microorganisms (PSM) can improve P availability by mobilizing inorganic phosphate and mineralizing organic phosphorus, thereby contributing to soil P cycling and plant P acquisition [68,69]. In our study, NYN-1 produced clear halos on both inorganic phosphate-solubilizing and organic phosphorus-mineralizing media (Table 1; Figure 2b,c), consistent with a phosphate-mobilizing phenotype. Genome annotation further identified multiple genes that may underlie this trait (Figure 6; Table 2), including *phoA* (alkaline phosphatase) and genes associated with phosphodiester/phosphonate turnover, such as *glpQ*, *phnW*, and *phnX* [33,34,35]. These genes may support hydrolysis of phosphate monoesters and diesters, as well as the utilization of diverse organic phosphorus substrates.

Notably, genes typically associated with PQQ-dependent gluconic acid biosynthesis (*pqqA*–*pqqF*), a pathway frequently linked to inorganic phosphate solubilization via organic-acid-mediated acidification, were not detected in the NYN-1 genome [70]. In contrast, NYN-1 harbored genes involved in gluconate transport and metabolism (e.g., *gntP*, *gntK*, and *gnd*), suggesting potential for gluconate utilization and broader organic-acid metabolism rather than a canonical PQQ–gluconic acid production route. Together, these observations imply that NYN-1 may mobilize inorganic phosphate through PQQ-independent mechanisms and/or secretion of alternative organic acids.

Potassium is an essential nutrient for plant growth and plays important roles in metabolism, including cellular biosynthesis, enzyme activity, and the production of proteins and vitamins. Potassium-solubilizing bacteria typically release K^+^ by secreting organic acids such as citric acid, oxalic acid, tartaric acid, and succinic acid, and further enhance the dissolution of potassium compounds through proton release and complexation with metal ions such as Fe^2+^, Al^3+^, and Ca^2+^ [71]. The NYN-1 genome contains genes encoding acetate kinase and malate dehydrogenase, suggesting that insoluble potassium minerals may be solubilized through the secretion of acetate and malate.

Genome annotation did not identify nitrogenase-encoding genes in NYN-1, consistent with the absence of nitrogen fixation activity in qualitative screening (Table 1). Nevertheless, NYN-1 showed NH_4_^+^ production in peptone water (Figure 2a; Table 1), and its genome harbors *gudB*, encoding glutamate dehydrogenase, which can release NH_4_^+^ during the conversion of L-glutamate to α-ketoglutarate (Table 3). In addition, antiSMASH analysis identified a siderophore-related biosynthetic gene cluster in NYN-1 (cluster 3; Table 5; Figure 7b), suggesting genetic potential for siderophore-mediated iron acquisition. Given that iron availability is frequently limited in alkaline and salt-affected soils, this trait may enhance rhizosphere competitiveness and indirectly improve plant nutrition [72]. Subsequent research should quantify ammonium release and siderophore production under saline–alkaline conditions, using methods such as the CAS assay and iron-limited growth tests, to validate these proposed functions.

## 5. Conclusions

In this study, a native, moderately halophilic and alkali-tolerant bacterium, *Staphylococcus succinus* NYN-1, was isolated from the rhizosphere of the halophyte *Suaeda dendroides* in Xinjiang and taxonomically assigned based on phenotypic and genomic evidence. Integrating phenotypic assays with whole-genome sequencing and annotation, we identified genomic features consistent with adaptation to saline–alkaline stress, including multiple Na^+^/H^+^ antiporter genes/putative antiporter components and transporter systems potentially involved in compatible-solute uptake (e.g., glycine betaine, proline, glutamate, and trehalose). Genome analysis further indicated genetic potential for multiple plant growth-promoting functions, including phosphate mobilization (organic phosphorus mineralization and inorganic phosphate solubilization), potassium solubilization, NH_4_^+^ production, and siderophore-related iron acquisition. Pot experiments under 300 mM NaCl showed that NYN-1 inoculation mitigated salt-induced growth inhibition and improved growth performance of maize, cotton, and sunflower. Collectively, these findings suggest that *S. succinus* NYN-1 is a promising native halophilic PGPR candidate for saline–alkaline agricultural systems and provides a microbial resource for developing and further validating inoculant strategies in salt-affected soils.

## Figures and Tables

**Table 1:** Qualitative screening results for the plant growth-promoting activities of *S. succinus* NYN-1.

Plant Growth-Promoting Activity	Result
Inorganic phosphate solubilization	+
Organic phosphorus mineralization	+
Ammonium production	+
Potassium solubilization	+
Nitrogen fixation	−
IAA production	−
ACC deaminase activity	−

“+” indicates the presence of the corresponding activity, whereas “−” indicates its absence.

**Table 2:** Classification of phosphorus metabolism genes in *S. succinus* NYN-1 according to the three core functional categories of the soil microbial phosphorus cycle.

Functional Category	Gene ID	Gene Name	Gene Description
P Activation	gene0591	*phoA*	alkaline phosphatase
	gene1922	*glpQ*	glycerophosphodiester phosphodiesterase
	gene2570	*phnX*	phosphonoacetaldehyde hydrolase
	gene2571	*phnW*	2-aminoethylphosphonate—pyruvate transaminase
	gene0428	*gntK*	gluconokinase
	gene0136	*gntP*	gluconate:H+ symporter
	gene1360	*gnd*	NADP-dependent phosphogluconate dehydrogenase
	gene0228	*ppdK*	pyruvate, phosphate dikinase
	gene0756	*deoB*	phosphopentomutase
P Uptake	gene1471	*pstS*	PstS family phosphate ABC transporter substrate-binding protein
	gene1473	*pstA*	phosphate ABC transporter permease PstA
	gene1472	*pstC*	phosphate ABC transporter permease subunit PstC
	gene1474	*pstB*	phosphate ABC transporter ATP-binding protein PstB
	gene2144	*pit*	Inorganic phosphate transporter
	gene0056	*ugpC*	sn-glycerol-3-phosphate ABC transporter ATP-binding protein UgpC
	gene0056	*ugpC*	sn-glycerol-3-phosphate ABC transporter ATP-binding protein UgpC
	gene2144	*pit*	Inorganic phosphate transporter
	gene0056	*ugpC*	sn-glycerol-3-phosphate ABC transporter ATP-binding protein UgpC
Regulation of P-deficiency-induced Responses	gene0023	*phoP*	two-component response regulator PhoP
	gene1117		
	gene0546	*phoB*	two-component response regulator PhoB
	gene1437		
	gene1118	*phoR*	Alkaline phosphatase synthesis sensor protein PhoR
	gene1475	*phoU*	Phosphate-specific transport system accessory protein PhoU
	gene0426	*spoT*	GTP pyrophosphokinase family protein
	gene1235	*phoH*	PhoH family protein

**Table 3:** Genes putatively associated with NH_4_^+^ production and potassium solubilization in the genome of *S. succinus* NYN-1.

Functional Category	Gene ID	Gene Name	Gene Description
Ammonium Production	gene1923	*gudB*	NAD-specific glutamate dehydrogenase
	gene2253		
Potassium Solubilizing	gene1098	*ackA*	acetate kinase malate dehydrogenase
	gene2114	*mdh*	

**Table 4:** Transporter genes related to saline–alkaline tolerance in the genome of *S. succinus* NYN-1.

Gene ID	Gene Name	TCDB Description
gene0362	*nhaC*	Na^+^/H^+^ antiporter (Sodium/proton antiporter)—*Bacillus firmus*
gene0579		Na^+^/H^+^ antiporter nhaC OS = *Staphylococcus aureus* subsp. aureus
gene0612		HYPOTHETICAL NA+/H+ ANTIPORTER IN ANSB-SPOIIM INTERGENIC REGION—*Bacillus subtilis*
gene2181	*mnhA*	Na^+^/H^+^ antiporter subunit A—*Bacillus subtilis*
gene2180	*mnhB*	Na^+^/H^+^ antiporter OS = *Bacillus halodurans*
gene1930		Na^+^/H^+^ antiporter subunit B—*Staphylococcus aureus*
gene1931	*mnhC*	Na^+^/H^+^ antiporter subunit C—*Staphylococcus aureus*
gene2179		Na^+^/H^+^ antiporter OS = *Bacillus halodurans*
gene1932	*mnhD*	Na^+^/H^+^ antiporter subunit D—*Staphylococcus aureus*
gene2178		Na^+^/H^+^ antiporter subunit D—*Bacillus subtilis*
gene2177	*mnhE*	Na^+^/H^+^ antiporter OS = *Bacillus halodurans*
gene1933		Na^+^/H^+^ antiporter subunit E—*Staphylococcus aureus*
gene1934	*mnhF*	Na^+^/H^+^ antiporter subunit F—*Staphylococcus aureus*
gene2176		Na^+^/H^+^ antiporter subunit F—*Bacillus subtilis*
gene1935	*mnhG*	Na^+^/H^+^ antiporter subunit G—*Staphylococcus aureus*
gene2175		Na^+^/H^+^ antiporter subunit G—*Bacillus subtilis*
gene0473	*opuA*	Glycine betaine/carnitine/choline transport ATP-binding protein opuCA—*Bacillus subtilis*
gene0474	*opuBD*	Glycine betaine/carnitine/choline transport system permease protein opuCB—*Bacillus subtilis*
gene0476		Glycine betaine/carnitine/choline transport system permease protein opuCD—*Bacillus subtilis*
gene0475	*opuC*	Glycine betaine/carnitine/choline-binding protein precursor (Osmoprotectant-binding protein)—*Bacillus subtilis*
gene1035		
gene0695	*opuD*	glycine betaine transporter OpuD—*Staphylococcus aureus*
gene1053		GLYCINE BETAINE TRANSPORTER OPUD—*Bacillus subtilis*
gene1548		
gene0515	*gltP*	PROTON/SODIUM-GLUTAMATE SYMPORT PROTEIN (GLUTAMATE-ASPARTATE CARRIER PROTEIN)—*Bacillus stearothermophilus*
gene0562	*gltS*	Glutamate/sodium ion symporter, GltS OS=*Pseudomonas aeruginosa*
gene2380	*treB*	Probable PTS system, trehalose-specific IIBC component—*Bacillus subtilis*

**Table 5:** Predicted secondary metabolite biosynthetic gene clusters in the *S. succinus* NYN-1 genome.

Cluster ID	Type	MIBiG Accession	Similar Cluster	Similarity (%)	Gene No.
cluster1	NRPS	-	-	-	39
cluster2	T3PKS	BGC0000758	capsular polysaccharide	3	34
cluster3	siderophore	BGC0000944	staphyloferrin A	100	12
cluster4	cyclic-lactone-autoinducer	BGC0000082	kijanimicin	4	18
cluster5	terpene	-	-	-	26
cluster6	terpene	-	-	-	25

## Data Availability

The complete genome sequence of strain NYN-1 has been deposited in the NCBI GenBank database under the accession number JBTWDY000000000.1.
